# Massive pulmonary embolism complicating mild Covid 19 pneumonia: Successful systemic thrombolysis using rt-PA in an elderly patient: A case report

**DOI:** 10.1016/j.amsu.2021.103090

**Published:** 2021-11-27

**Authors:** Saîda Amaqdouf, Zakariae Belarbi, Mustapha Beghi, Chaimae Toutai, Nabila Ismaili, Noha El Ouafi

**Affiliations:** a Department of Cardiology, Mohammed VI University Hospital of Oujda, Mohammed First University of Oujda, Morocco; b Laboratory of Epidemiology, Clinical Research and Public Health, Faculty of Medicine and Pharmacy, Mohammed the First University of Oujda, Morocco

**Keywords:** Covid 19, Hypercoagulability, Massive pulmonary embolism, Pulmonary embolism, Thromboembolic disease, Right heart failure, Thrombus, rt-PA, Elderly, Case report

## Abstract

**Introduction:**

and importance: Pulmonary embolism (PE) is still a major health concern around the world, and its link with SARS Cov-2 has led to an increase in morbidity, mortality, and ICU hospitalizations.

**Case presentation:**

We present the case of a 92-year-old man with no prior medical history who admitted to our hospital in a state of acute respiratory failure, echocardiography revealed an acute right heart syndrome with a thrombus in the right atrium, computed tomography pulmonary angiogram revealed bilateral massive pulmonary embolism as well as Covid-19 pneumonia. He was treated with systemic thrombolysis using intravenous rt-PA (recombinant tissue plasminogen activator) with immediate clinical improvement and no hemorrhagic complications.

**Clinical discussion:**

In the presence of the SARs Cov-2 infection, several reports have indicated considerable procoagulant events, including life-threatening pulmonary embolism. There are still no current guidelines for the treatment of VTE in COVID-19 patients, but they are largely consistent with non-COVID-19 recommendations. Elderly patients are considered to be at high risk of developing thromboembolic complications, and also and above all are vulnerable to bleeding complications from anticoagulant treatments.

**Conclusion:**

This case highlight the importance of considering thromboembolic complications despite the severity of the associated SARS-cov-2 pneumonia and the role of prophylactic anticoagulation for Covid-19 patients hospitalized or not.

## Highlights

• COVID-19 is associated with an increased risk for venous thromboembolism (VTE).
• Despite the severity of the Covid19 infection, it is necessary to consider and prevent thromboembolic complications.
• Existing guidelines for the management of pulmonary embolism appears to be applicable to COVID-19 patients.
• Elderly patients pose important challenges in the management of PE, from its suspicion to its diagnosis and treatment.

## 1 Introduction

Since its first appearance in Wuhan-China in December 2019, the SARS Covid 2 infection has been known as a life-changing pandemic affecting especially the public health and the economic field. Covid −19 infection was first considered to be a highly contagious respiratory infection, then as a multi-systemic disease with multiple manifestations. (renal, cardiac, thromboembolic, nervous, cutaneous, and gastrointestinal) [1].

COVID-19 infection triggers an immunological hyper-response, which is linked to hypercoagulability and a significant risk of venous thromboembolism (VTE) [2]. Several studies have attempted to explain the link between Covid 19 infection and coagulation abnormalities, this prompted questions and debate over the best technique for VTE prophylaxis and anti-thrombotic medication. Although there are no current guidelines for treating VTE in COVID-19 patients, they are essentially in line with non-COVID-19 recommendations [3].

Here we report a case of a 92-year-old man with a massive pulmonary embolism caused by a Covid-19 infection that was discovered by chance on CTPA and successfully treated with systemic thrombolysis using rt-PA without complications.

### 1.1 Patient information

A 92-year-old man with no previous medical history who was admitted to the emergency department for sudden dyspnea, cyanosis, and severe fatigability. No fever, cough, anosmia, or other possible sign of Covid-19 infection was reported in the days preceding his admission, and none of his family members were symptomatic or suspected of having Covid-19 virus infection. On admission, the clinical examination revealed a confused and short of breath patient in a state of shock, as vital signs were as follow: blood pressure was 80/50 mmHg, heart rate was 112 bpm, respiratory rate was 28 breaths per minute and oxygen saturation was 65% on room air. Physical examination revealed an edematous, cyanotic left lower limb with a positive Homans' sign and absence of peripheral pulses; according to the family, this condition of the lower limb had lasted progressively for at least 10 days.

**Fig. 1:**
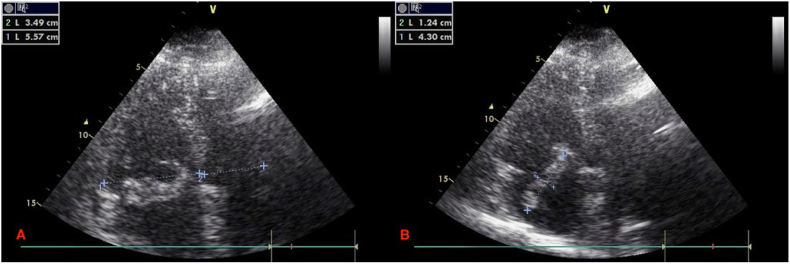
TTE showing right ventricular strain**;** A: dilated right ventricular (VD/VG: 1.6) B: thrombus in the right atrium.

**Fig. 2:**
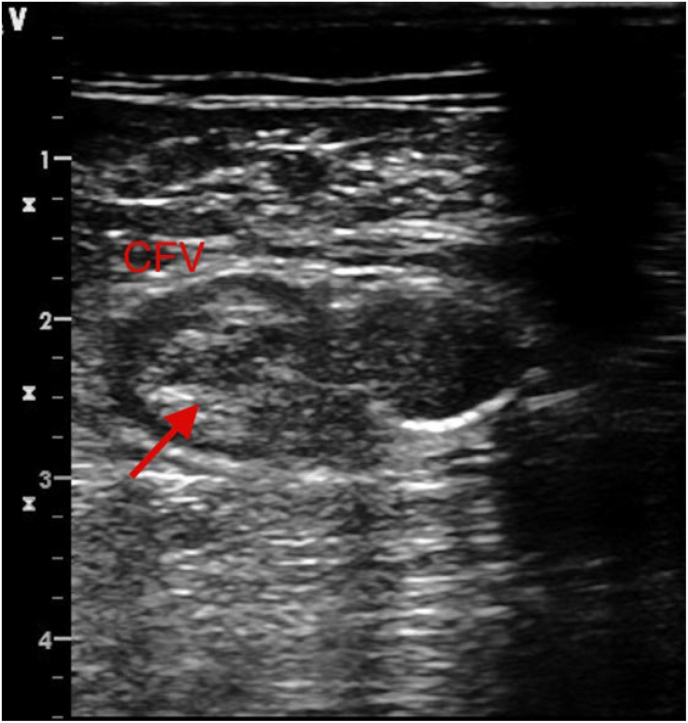
Venous Doppler ultrasound showed a thrombus in the left common femoral vein.

**Fig. 3:**
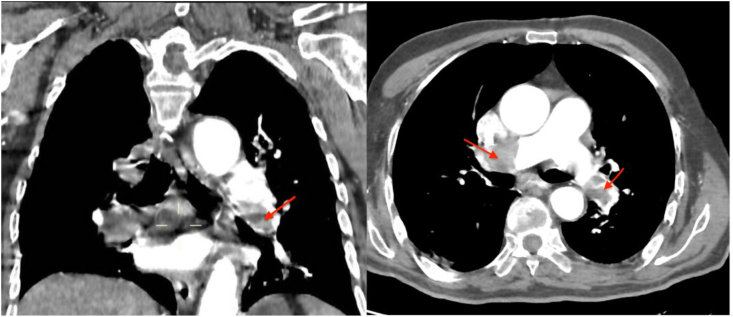
A: Axial image from the CT pulmonary angiogram (CTPA) demonstrates bilateral proximal pulmonary emboli B: Coronal image from the CT pulmonary angiogram.

**Fig. 4:**
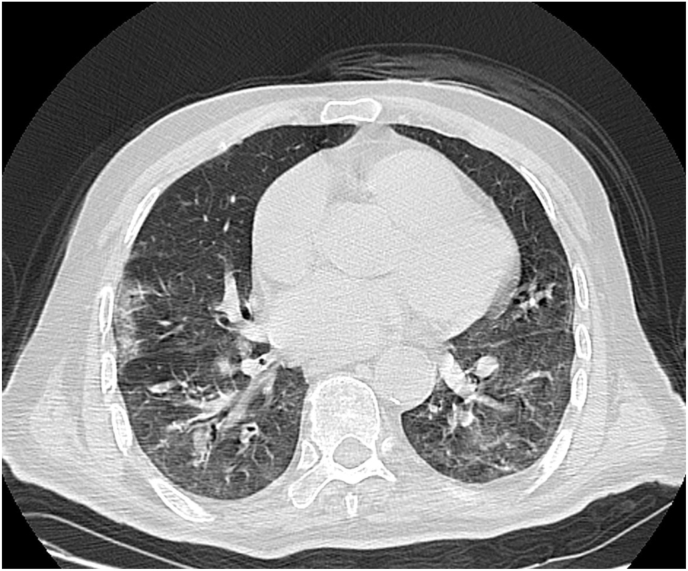
Axial image (lung windows) from the CT pulmonary angiogram demonstrating extensive multifocal patchy ground-glass opacities in lung parenchyma.

While the patient was receiving oxygen-therapy via a high-flow mask, bedside transthoracic echocardiography revealed a thrombus in the right atrium (43 × 12 mm), dilated right ventricular (VD/VG: 1.6) in systolic dysfunction (Tricuspid annular plane systolic excursion:12mm, peak systolic velocity of annulus tricuspid: 7cm/s) associated with severe pulmonary hypertension (67 mmHg) and abnormal septum motion and dilated pulmonary artery trunk (29 mm) [Fig. 1]. The left ventricular ejection fraction was around 55%. Venous Doppler ultrasound showed a thrombus in the left common femoral vein **[**Fig. 2**].** The CTPA was immediately performed, which confirmed bilateral massive pulmonary embolism **[**Fig. 3**]**, lung windows demonstrate peripheral patchy ground-glass opacities consistent with SARS-CoV-2 pneumonia with an estimation of only 25% of lung damage**[**Fig. 4**].**

Laboratory work revealed significant elevated C-reactive protein at 161 mg/l, white blood cell count of 24,610/μl, hemoglobin at 15/dl, elevated lactate dehydrogenase (1536U/L), elevated liver enzymes with ALT 727U/L and AST 429 U/L, troponin I was elevated to 317 ng/mL and PCR SARS-Cov2 was positive.

According to his clinical state, echocardiography and scanner findings and after checking the absence of any contraindications, the patient was immediately put on systemic thrombolysis using rt-PA with the following protocol: 100 mg IV infusion pump over 2 hours. The hemodynamic and neurologic status of the patient were being closely monitored, the procedure was expeditiously accomplished without any bleeding, oxygen saturation was gradually increasing to 89% with 6 L of oxygen, blood pressure raised to 100/60 mmHg, the patient was transferred to the ICU to continue assessments and begin unfractionated heparin infusion along with antibiotics.

Follow up and outcome:

Day 3: The patient was no longer dyspneic, with oxygen saturation at 95% under 4 L of oxygen per minute and blood pressure at 120/70 mmHg, and the physical examination showed a significant improvement of the blue phlebitis, notably a marked regression of the volume of the left leg with resumption of the perfusion of the peripheral arteries.

Day 5: In breathing ambient, oxygen saturation was 94%.

Day 8: He was discharged from the hospital on Dabigatran 110 mg twice a day along with Covid −19 treatment.

Day 30: The patient returned for consultation 30 days after being discharged; he was fine and eupneic, and the TTE revealed a slightly dilated right ventricle with good systolic function and no thrombus.

## 2 Discussion

Infection with the SARS-CoV-2 virus has been linked to aberrant coagulation parameters, particularly high D-dimer levels, which are linked to a poor prognosis [4]. Over the last year, many scientific studies and reviews aimed to better understand and elucidate the association between SARS Covid-2 infection and thromboembolic disease.

Unlike sepsis intravascular coagulopathy and disseminated intravascular coagulopathy, which are manifestations of systemic coagulopathy, the new term PIC (pulmonary intravascular coagulopathy) is a manifestation of a local coagulation disorder in the lung specific to SARS-Covid 2, it was described as a MAS-like state (macrophage activation syndrome) that activates extensive pulmonary immunothrombosis [5,6].

Micothrombis were found in pulmonary capillaries in numerous post-mortem investigations [7]. A prospective cohort analysis based on autopsy results of 12 COVID-19 patients found a significant incidence of deep venous thrombosis (58%) and pulmonary embolism as the direct cause of death in one-third of the patients [8].

Pulmonary embolism (PE) alone remains until nowadays a worldwide major health concern, It's association with the SARS Covid-2 increase in morbidity, mortality and ICU admissions. Referring to a systematic review and meta-analysis the incidence of COVID-19 patients developing PE was 15.3% and mortality rate was 45.1% [9].

High-risk PE defined as the presence of cardiogenic shock, represents the most severe manifestation of venous thromboembolic disease, it is associated with high rate of mortality the moment or within few hours after diagnosis even after initiating reperfusion treatments. According to ESC guidelines, systemic thrombolysis is the treatment of choice for patients with high-risk PE, however, if thrombolysis is contraindicated, surgical pulmonary embolectomy or percutaneous catheter-directed therapy are further alternatives for reperfusion [10]. Also It is recommended that anticoagulation must be initiated without delay in patients with high-risk PE [10]. IV unfractionated heparin (UFH) is the preferred anticoagulant for those receiving alteplase for PE before alteplase infusion [11]. However, guidelines for management of EP in the context of COVID-19, are not yet in place, whereas and given the frequency of acute PE in COVID-19 patients, based on the existing data, preventive anticoagulation should be started as soon as possible to avoid thrombotic events [12].

According to published case reports and small case series, all reperfusion means have been tried to treat massive PE in Covid-19 patients, of all 24 cases, 15 patients (62,5%) Treated by system thrombolysis 2 of them presented hemorrhagic complications and 4 did not survived. 5 patients (20%) successfully treated by catheter-directed thrombolysis, and 4 patients underwent mechanical embolectomy, one of them did not survived due to septic shock. The high-risk PE induced by SARS cov-2 was reported especially in patients admitted with severe or at least moderate Covid-19 pneumonia.

The particularity of our case is that our patient is 92 years old witch make him the oldest reported patient with massive PE and COVID-19, he was admitted in a hypoxic choc state which was most likely due to high risk PE rather than the Covid-19 pneumonia, causing RV dysfunction early detected by bedside ETT, the CTPA confirmed the diagnosis of massive PE along with Covid-19 pneumonia with only 25% of lung damage. We treated our patient with systemic thrombolysis, surely the risk of bleeding should be not underestimated, specially in our patient due to his age, nevertheless the patient was hemodynamically unstable with life threatening PE which qualified him to urgent reperfusion therapy. Given his age, the physical activity could be sufficiently reduced to cause a state of hypercoagulability, so another unknown underlying cause could be at the origin of the occurrence of his PE.

Elderly patients are not only at a higher risk of venous thromboembolism, but they are also at a higher risk of unfavorable clinical outcomes and treatment-related complications.

Our case has been reported in line with THE SCARE 2020 criteria [13].

## 3 Conclusion

In the context of SARs COV-2 infection, the occurrence of high-risk EP is considered a management challenge, given the lack of clear consensus, and given that PE could be related to other unknown predisposing risk factors other than Covid 19.

The SARS-cov-2 infection is now considered as a potential thromboembolic risk factor, this case highlight the importance of considering thromboembolic complications despite the severity of the associated pneumonia and the role of prophylactic anticoagulation for Covid-19 patients hospitalized or not. Further research and larger-scale studies are needed to accurately estimate the incidence and mortality of PE in patients with SARS-Cov-2 disease, as well as the optimal treatment regimens.

## Provenance and peer review

Not commissioned, externally peer-reviewed.

## Ethical approval

The ethical committee approval was not required give the article type (case report). However, the written consent to publish the clinical data of the patients was given and is available to check by the handling editor if needed.

## Sources of funding

None.

## Author contribution

Saîda Amaqdouf: Study concept, Data collection, Data analysis, Writing the paper.: Data analysis, Contributor. Zakariae Belarbi: Data analysis, Contributor. Mustapha Beghi: Data analysis, Contributor, Chaimae Toutai: Data analysis, Contributor. Nabila Ismaili: Supervision and data validation. Noha El Ouafi: Supervision and data validation.

## Trial registry number

This is not an original research project involving human participants in an interventional or an observational study but a case report. This registration is not applicable.

## Guarantor

Saîda Amaqdouf.

## Consent

Written informed consent was obtained from the patient for publication of this case report and accompanying images.

## Declaration of competing interest

The authors declare no competing interest.
